# Swine hemorrhagic shock model and pathophysiological changes in a desert dry-heat environment

**DOI:** 10.1371/journal.pone.0244727

**Published:** 2021-01-05

**Authors:** Caifu Shen, Dunhong Wei, Guangjun Wang, Yan Kang, Fan Yang, Qin Xu, Liang Xia, Jiangwei Liu

**Affiliations:** 1 Key Laboratory of Special Environmental Medicine of Xinjiang, General Hospital of Xinjiang Military Command of the Chinese People’s Liberation Army, Urumqi, China; 2 The 69240 Army Hospital of the Chinese People’s Liberation Army, Xinjiang, Urumqi, China; National Veterinary School of Toulouse, FRANCE

## Abstract

**Background:**

This study aimed to establish a traumatic hemorrhagic shock (THS) model in swine and examine pathophysiological characteristics in a dry-heat environment.

**Methods:**

Forty domestic Landrace piglets were randomly assigned to four study groups: normal temperature non-shock (NS), normal temperature THS (NTHS), desert dry-heat non-shock (DS), and desert dry-hot THS (DTHS) groups. The groups were exposed to either normal temperature (25°C) or dry heat (40.5°C) for 3 h. To induce THS, anesthetized piglets in the NTHS and DTHS groups were subjected to liver trauma and hypovolemic shock until death, and piglets in the NS and DS groups were euthanized at 11 h and 4 h, respectively. Body temperature, blood gas, cytokine production, and organ function were assessed before and after environmental exposure at 0 h and at every 30 min after shock to death. Hemodynamics was measured post exposure and post-shock at 0 h and at every 30 min after shock to death.

**Results:**

Survival, body temperature, oxygen delivery, oxygen consumption, and cardiac output were significantly different for traumatic hemorrhagic shock in the dry-heat groups compared to those in the normal temperature groups. Lactic acid and IL-6 had a marked increase at 0.5 h, followed by a progressive and rapid increase in the DTHS group.

**Conclusions:**

Our findings suggest that the combined action of a dry-heat environment and THS leads to higher oxygen metabolism, poorer hemodynamic stability, and earlier and more severe inflammatory response with higher mortality.

## Introduction

Trauma hemorrhagic shock (THS) is a type of hypovolemic shock involving massive blood loss that results in a pathologic state in which intravascular volume and oxygen delivery (DO2) are impaired. This can lead to inadequate tissue perfusion along with cellular hypoxia and metabolic disorders [1, 2]. The WHO estimates that more than 5 million people die each year from trauma, accounting for 9% of the global death toll [3, 4]. Another report indicates that from October 2001 to June 2011, 4596 people died in the Iraq war from traumatic hemorrhagic shock, and 87.3% of them died before reaching the hospital [5]. Iraq has an extremely hot and dry climate, with variations in temperature and intense ultraviolet radiation [6], and our experimental team's previous research showed that the post-traumatic mortality rate of rats in the dry-heat desert environment is also high [7]. Therefore, we speculated that the high mortality rate in the Iraq war was related to the dry-heat environment.

We are currently facing global warming and environmental changes. Global average temperatures increased by approximately 0.8°C in the last century, and global surface temperatures are rising [8–11]. By the end of this century, the average temperature could rise by 3–4°C [12]. The rise in temperature may have a greater effect on desert climate areas. For example, during the summer of 2015, Iran’s thermal index reached a value higher than the world record of 74°C (165°F) [13]. At approximately 48 million square kilometers, the world’s desert climate area accounts for one-third of the world’s land area and is still increasing. Previous studies have focused on either the treatment or mechanism of THS or simple heat stress but not on both complex factors combined. We have conducted several studies on desert dry-heat environments [6, 7, 14, 15]. However, to date, no study has used large-scale animal models to investigate the pathophysiological characteristics of THS in a desert dry-heat environment. Therefore, we established a THS model in swine and investigated pathophysiological characteristics in a dry-heat environment to explain the probable mechanisms of the combined stresses.

## Methods

This animal study was approved by the Animal Committee of the General Hospital of Xinjiang military area of the People's Liberation Army of China (Protocol Number: WDLL2014007) and was conducted in accordance with appropriate AAALAC guidelines(https://www.aaalac.org/). All surgeries were performed under anesthesia, and all efforts were made to minimize suffering.

### Animal housing and husbandry

All pigs were kept separately in single cages (Large Animal Breeding Room, Department of Animal Experiment, General Hospital of Xinjiang Military Region, China) to better monitor their health individually and to ensure that they do not cause harm to each other. Temperature was maintained at 24°C–26°C, while humidity was maintained at 30%–40% (the feeding environment of the animal provider). Animal care and experiments were conducted according to the National Science Council guidelines (https://doi.org/10.1093/ilar/ilw018). Swine were fed with a standard diet (13.6 MJ/kg, Zizhubao, Zhengda Feed, China) and observed for ≥1 week to ensure acclimatization. In addition, all animals underwent fasting for 24 h before surgery but were allowed water ad libitum until 4 h before the experiment.

### Experimental settings

Forty male Landrace piglets (25–35 kg, age: approximately 9 weeks. Zhengda Animal Husbandry Co., Ltd. Xinjiang, China) were randomly assigned to the following four groups: the normal temperature non-shock group (NS, T: 25°C±1°C, dry-humidity: 35%±5%, n = 10), the normal temperature THS group (NTHS, T: 25°C±1°C, dry-humidity: 35%±5%, n = 10), the desert dry-heat non-shock group (DS, T: 40.5°C±0.5°C, dry-humidity: 10%±2%, n = 10), and the desert dry-heat THS group (DTHS, T: 40.5°C±0.5°C, dry-humidity: 10%±2%, n = 10), The surgical bed was not heated to simulate the natural course of shock in a desert dry-heat environment. Climate simulation experiments were performed in The Simulated Climate Cabin for Special Environment of Northwest of China [16]. Before an animal entered the cabin, it was gauged to meet each group’s relevant environmental requirement. As described above, all experimental animals were exposed to their respective environments for 3 h before receiving anesthesia.

### Anesthesia and instrumentation

After environmental exposure, animals were sedated with an intramuscular injection of ketamine (20 mg/kg) (Gutian Pharmaceutical Co., Ltd. Fujian, China) and atropine (0.05 mg/kg) (Jinyao Pharmaceutical Co., Ltd. Tianjin, China). Immediately afterward, animals were intubated with an orotracheal tube 7.5 mm in diameter. Positive pressure ventilation was initiated with a volume-controlled ventilator (EX-60 Mindray Biomedical Electronics Co., Ltd. Shenzhen, China) set to deliver a tidal volume of 8 ml/kg. The respiratory rate was adjusted at the beginning of the experiment to maintain the end-tidal PCO_2_ between 35 and 45 mmHg. After that, no adjustments in ventilation settings were made throughout the experiment. Anesthesia was maintained by subjecting the animals to continuous inhalation of sevoflurane (Hengrui Pharmaceutical Co., Ltd., Shanghai, China) and injection with fentanyl citrate (Yichang Renfu Pharmaceutical Co., Ltd., Hubei, China) (0.002 mg/kg) every 60 minutes. When spontaneous breathing or fasciculation occurred, vecuronium bromide (Xianju Pharmaceutical Co., Ltd. Zhejiang, China) was injected in a peripheral vein (0.03 mg/kg). The bispectral index (BIS) (target range, 40–60) was employed to monitor the dosage of anesthesia [17].

### Monitoring setup

The animals were placed on the operating table in the supine position. A continuous electrocardiographic monitor, a temperature sensor, and a pulse oximetry set were attached to the chest wall, rectum, and tail, respectively.

### Surgical preparation

**Fig 1:**
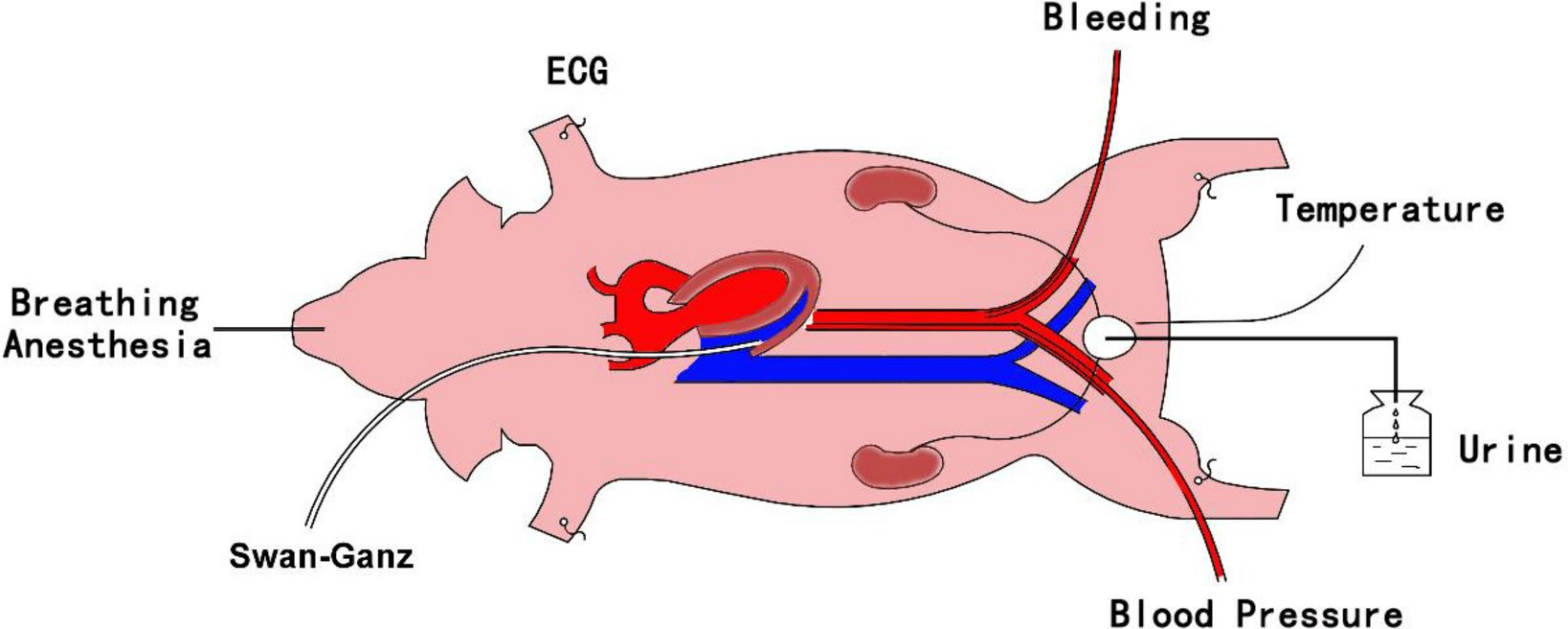
Schematic diagram of the model. ECG, electrocardiogram; MAP, mean arterial pressure.

In the supine position, approximately 5–10 cm incisions were made in both sides of the groin and the right side of the neck to expose and isolate both ends of the external iliac artery and the right external jugular vein. A 7-F catheter was advanced through the right external iliac artery into the descending thoracic aorta for continuous pressure measurement. An adult double lumen venous tube was inserted through the left external iliac artery and situated in the iliac artery for quick extraction of blood. A Swan-Ganz thermodilution catheter was advanced through the right jugular vein into the pulmonary artery to measure hemodynamics and collect blood samples. As the thermoregulatory center, the hypothalamus may play a particularly important role during temperature dysregulation in an organism. To avoid the artificial effect of hypothalamic thermoregulatory function, as much as possible, external carotid artery ligation or puncture was avoided throughout the whole experiment to avoid affecting the blood supply to the brain. The model diagram is shown in the Fig 1.

Next, we performed midline laparotomy. As pigs have a contractile spleen that is approximately three times larger than a human spleen [18], spontaneous contraction can occur following a hemorrhagic shock to auto-transfuse a significant volume of blood. Accordingly, to simulate physiological responses to hemorrhagic shock in humans, we performed splenectomy in the NTHS and DTHS groups. The spleen was immediately weighed, and the piglet was infused with warm lactated Ringer’s solution (Minsheng Pharma, Hangzhou, China) at three times the volume of the resected spleen to replace the blood volume contained in the removed spleen [18, 19]. A complete splenectomy and 1/4 resection of the left liver were performed to simulate liver trauma and complete hemostasis. We did not perform resection of the liver and spleen in the NS and DS groups because the two groups were not hemorrhagic shock models. The surgery and procedures conducted were under the appropriate individualized environmental conditions.

### Hemorrhagic shock protocol compliance

To complete the above surgery, we began to bleed the animals immediately. The pigs in NTHS and DTHS underwent hemorrhage to a mean arterial pressure (MAP) of 45±5 mmHg within 10 min, and this pressure was maintained for 20 min. The withdrawn blood was saved in sterile empty blood bags. The goal MAP (45±5 mmHg) was maintained during the hemorrhagic shock period, and animals received further hemorrhaging or reinfusion, as necessary, to maintain the goal pressure. Then, all bags and blood-soaked gauze were weighed, and the blood volume was calculated (blood density = 1.06 g/ml).

The completion point for the stable period (after MAP was maintained for 20 min) was shock 0 h. Until the natural death of the animals in the NTHS and DTHS groups, pumping blood samples were collected every 30 min, and body temperature, heart rate (HR), and blood pressure were recorded every 10 min. After the shock model was successfully implemented, none of the pigs required fluid resuscitation, had blood loss, or required other interventions.

### Experimental outcomes

The primary outcomes of the experiment were survival time, amount of bleeding, body temperature, HR, MAP, and cardiac output (CO). The secondary outcomes were plasma cytokines (interleukin [IL]-6, IL-10, IL-1β, and tumor necrosis factor-α [TNF-α]), arterial lactate (Lac), DO_2_, and oxygen consumption (VO_2_).

### Blood measurements

In each group, blood samples were collected from the external iliac artery and the pulmonary artery. Arterial and venous blood samples were used to measure blood gas parameters (GEM3000 Blood Gas Analyzer, Instrumentation Laboratory, America.), including arterial oxygen saturation (SaO2), venous oxygen saturation (SvO2), and lactate. DO_2_ and VO_2_ were calculated according to the following equations:

$${\text{DO}}_{2}=1.34\times \text{SaO}2\times \text{Hemoglobin}\;\left(\text{g}/\text{L}\right)\times \text{CO}\times 10$$

$${\text{VO}}_{2}=\left(\text{SaO}2—\text{SvO}2\right)/\text{SaO}2$$

Blood samples were also used to measure blood urea nitrogen, creatinine, alanine aminotransferase, and aspartate aminotransferase (AST) (BS-180 Automatic biochemical analyzer, Mindray Biomedical Electronics Co., Ltd, Shenzhen, China).

### Hemodynamic measurements

CO was measured using a thermistor of the Swan-Ganz thermodilution catheter. Cardiac index was assessed using the following equation: (CI) = CO/body surface area (0.073·body-weight^2/3^ [kg]). MAP and HR were measured using a T8 monitor (Mindray, Shenzhen, China).

### Cytokine measurements

We collected blood samples into tubes with separation gel and accelerator for testing. After centrifugation, serum samples were frozen at -20°C until analysis for TNF-α, IL-1β, IL-6, and IL-10 levels. All assays for cytokine measurement were performed in duplicate using a porcine cytokine ELISA assay kit (Anhui Joyee Biotechnics Co., Ltd, China). The experimental assays strictly followed the manufacturers’ recommended protocols. The duplicate results were averaged and used for statistical analysis.

### Statistical analysis

SPSS 23.0 (IBM Corp, Armonk, NY) was used for statistical analyses. Experimental results are expressed as mean ± standard deviation. Survival analysis was conducted using the Kaplan-Meier method. A multiple comparison test—post-hoc analysis-was used to compare the group differences at each time point. One-way analysis of variance was used for comparison within groups. P-values <0.05 were considered to be statistically significant.

## Results

No unexpected adverse events occurred during the experiment. The causes of death were THS and euthanasia (0.3% pentobarbital [Chemical Reagent Factory, Shanghai, China] 0.22 ml/kg intravenous).

**Fig 2:**
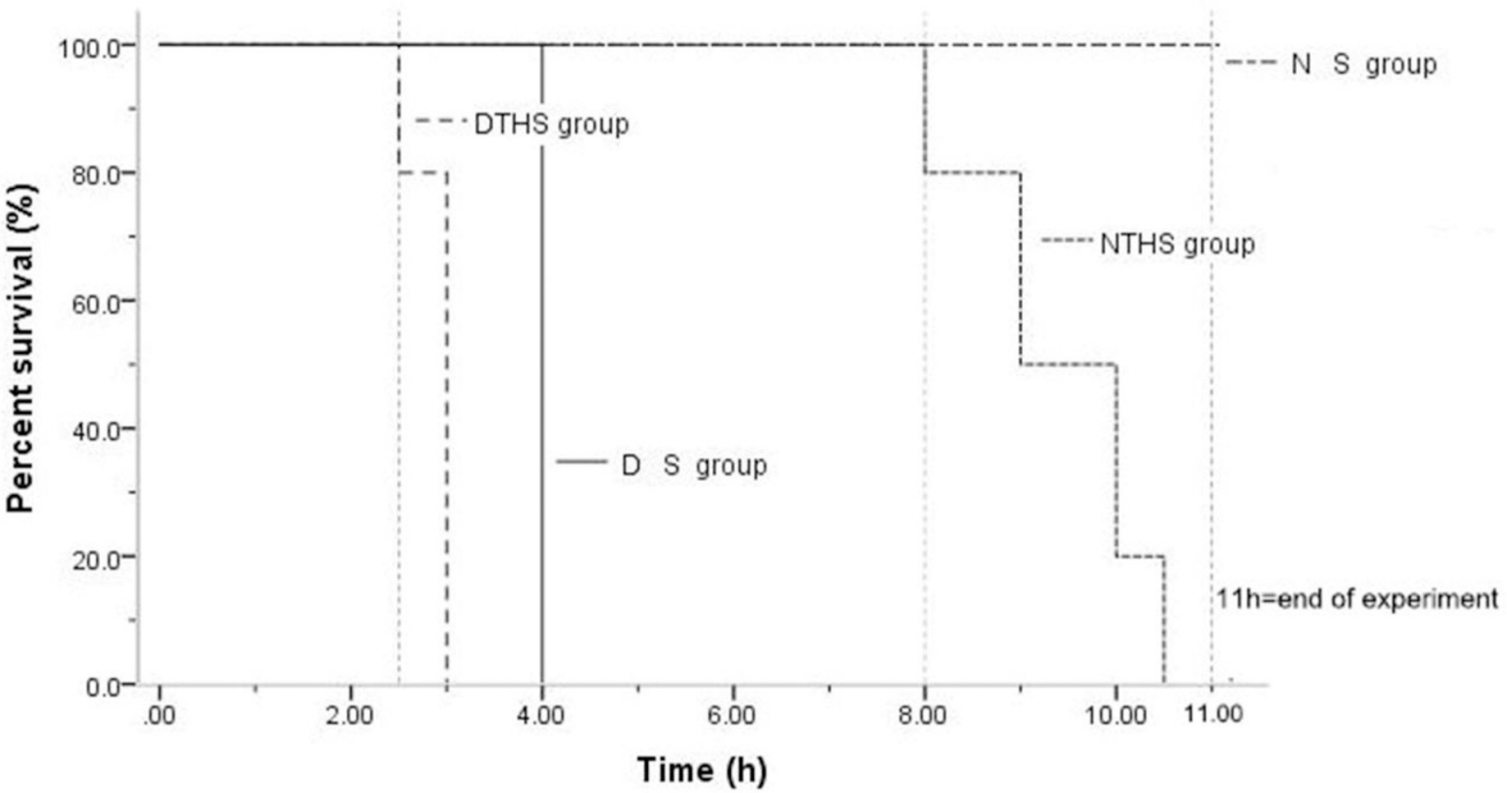
B: Survival curves in the NS, NTHS, DS, and DTHS groups. Dotted and broken line, NS group; dotted line, NTHS group; straight line, DS group; broken line, DTHS group.

The time and rate of survival for all groups are shown in Fig 1. For the NS group, no animal death was seen before 10.5 h. For the NTHS group, animal deaths began to occur at 8 h, and the longest survival time was ≤10.5 h. For the DS group, there was no animal death until 4 h. With a range of 2.5 h–3.5 h, the average survival time was only 3 h in the DTHS group, which was significantly shorter than in the DS and NTHS groups (P<0.001; Fig 2).

**Fig 3:**
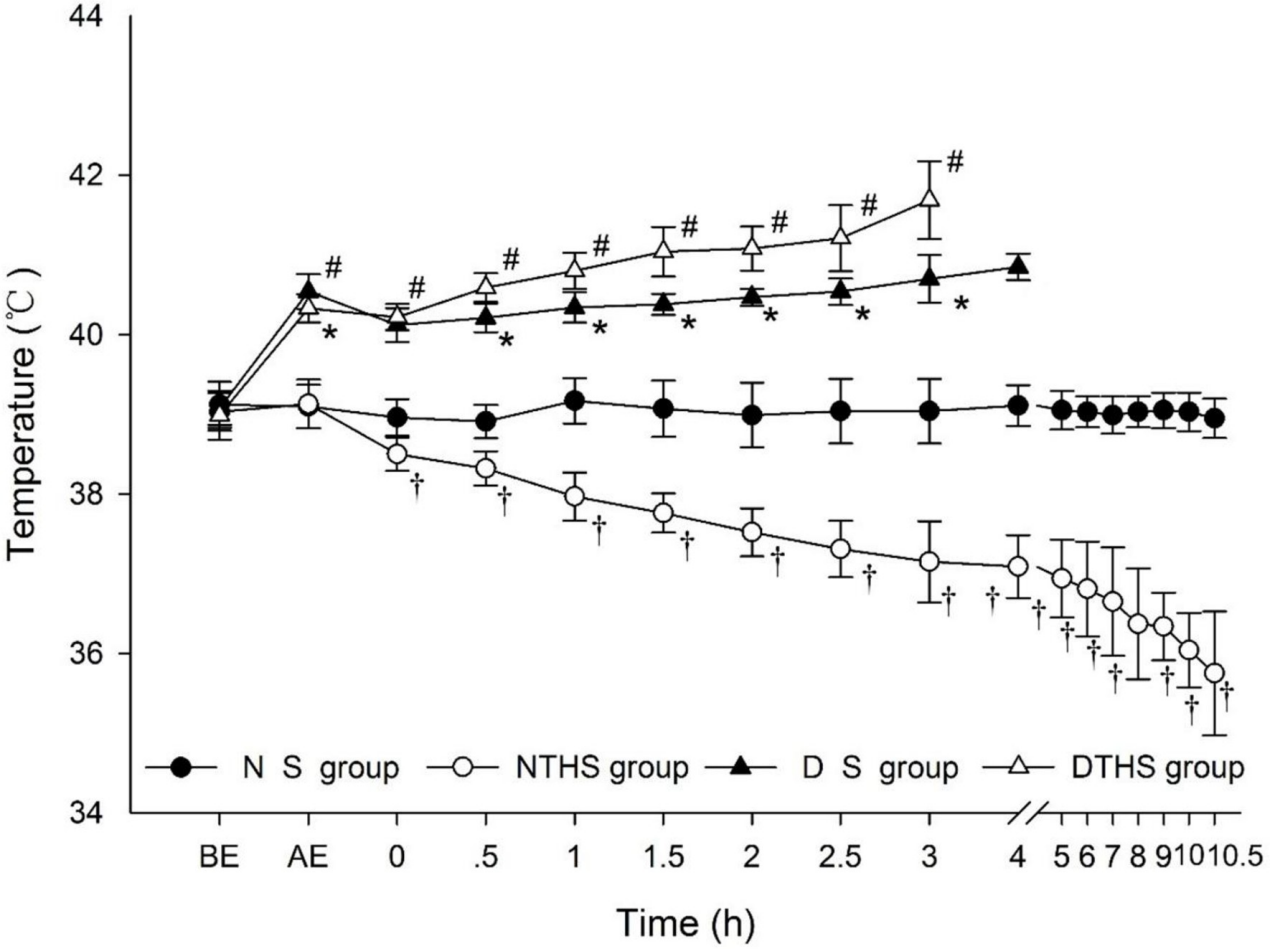
Temperature variation curve of desert dry-heat environment in traumatic hemorrhagic shock pigs. BE, before exposure; AE, after exposure. Values are shown as mean ± SEM. P<0.05 was considered statistically significant. ^†^P, ^#^P, and *P denote statistically significant difference for NS vs. NTHS, DTHS vs. NTHS, and DTHS vs. DS, respectively.

After 3 h of exposure, the temperature of animals in the dry-heat environment was significantly higher than that in the normal environment (P<0.001) (DS and DTHS versus NS and NTH). It is noteworthy that although temperature quickly declined after anesthesia for all observed subjects, it remained relatively stable in the NS group, and it gradually declined until the animals’ death in the NTHS group. In contrast, temperature steadily increased until death in the desert dry-heat environment groups. In the DS group, the average highest body temperature was lower than the ambient highest temperature, whereas in the DTHS group, the rapid rise in body temperature exceeded the highest ambient temperature (Fig 3).

**Fig 4:**
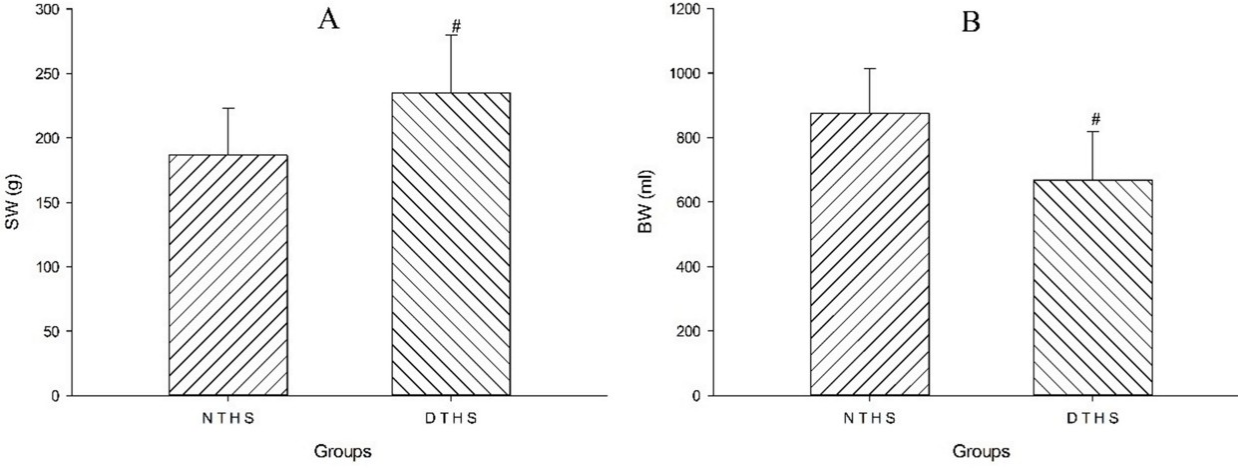
Comparison of volume of blood withdrawn and weight of spleen between NTHS and DTHS groups. A, weight of spleen; B, volume of blood withdrawn in two groups; BW, volume of blood withdrawn; SW, weight of spleen. Values are shown as mean ± SEM. P<0.05 was considered statistically significant.

The mean weight of the spleen in the DTHS group was significantly greater than that in the NTHS group (P<0.05; Fig 4A). The volume of blood withdrawn in the NTHS group was significantly larger than the volume withdrawn in the DTHS group (P<0.05; Fig 4B).

**Fig 5:**
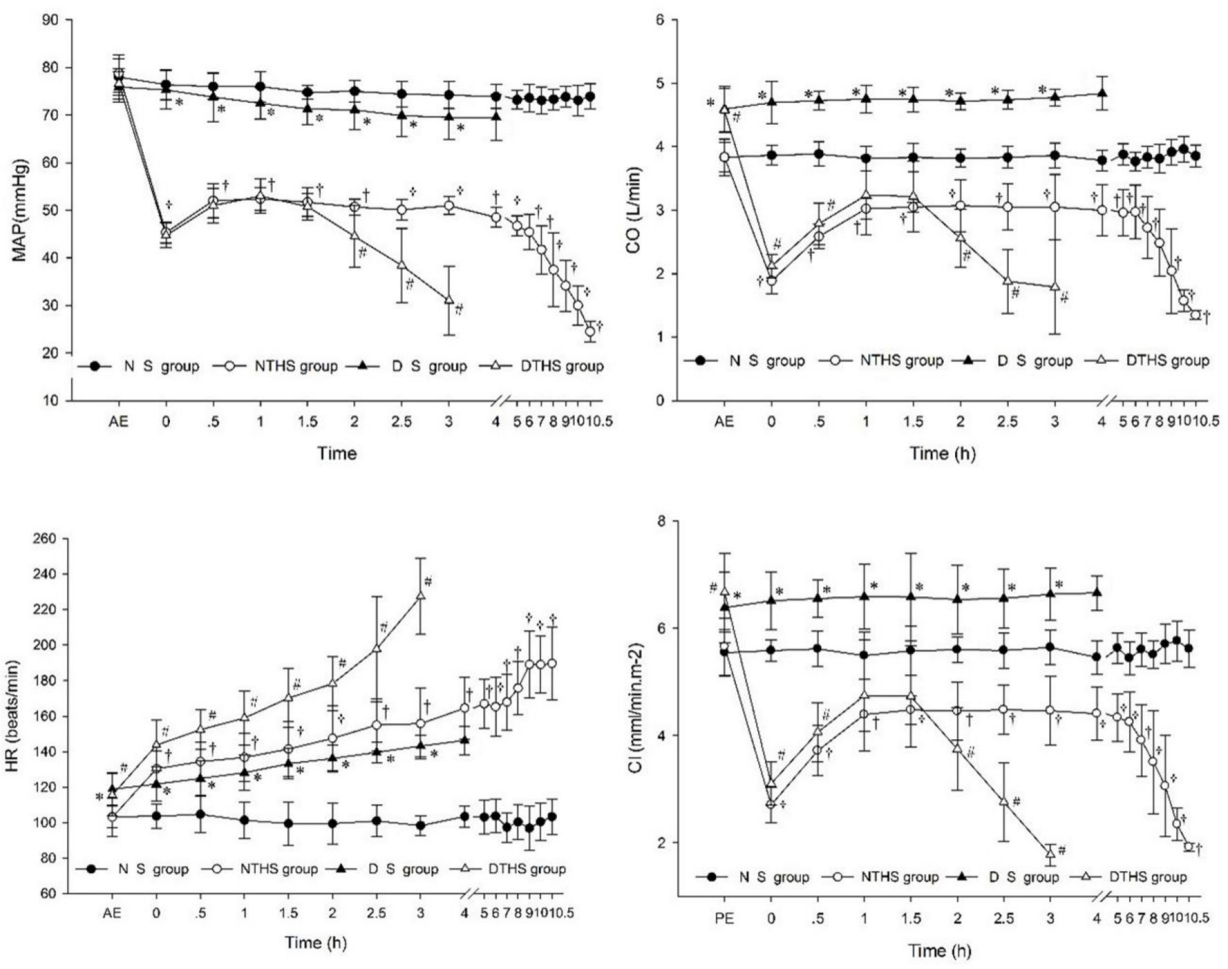
Hemodynamic change of the four groups. (a) MAP, mean arterial pressure; (b) CO, cardiac output; (c) HR, heart rate; (d) CI, cardiac index. Values are shown as mean ± SEM. P<0.05 was considered statistically significant. †P, #P, and *P denote statistically significant differences for NS vs. NTHS, DTHS vs. NTHS, and DTHS vs. DS, respectively.

All hemodynamic parameters were higher in subjects exposed to the dry-heat environment than in those exposed to the normal temperature environment, and there were significant differences in the parameters except for MAP. In the NTHS and DTHS groups, shock occurred with the rapid decrease of blood in the body; MAP, CO, and CI rapidly decreased. However, after stopping the loss of blood, the body went through a cycle of increasing, stabilizing, and decreasing MAP, CO, and CI until death. The HR increased for all groups except for the NS group. HR increased the earliest and fastest in the DTHS group (Fig 5).

**Fig 6:**
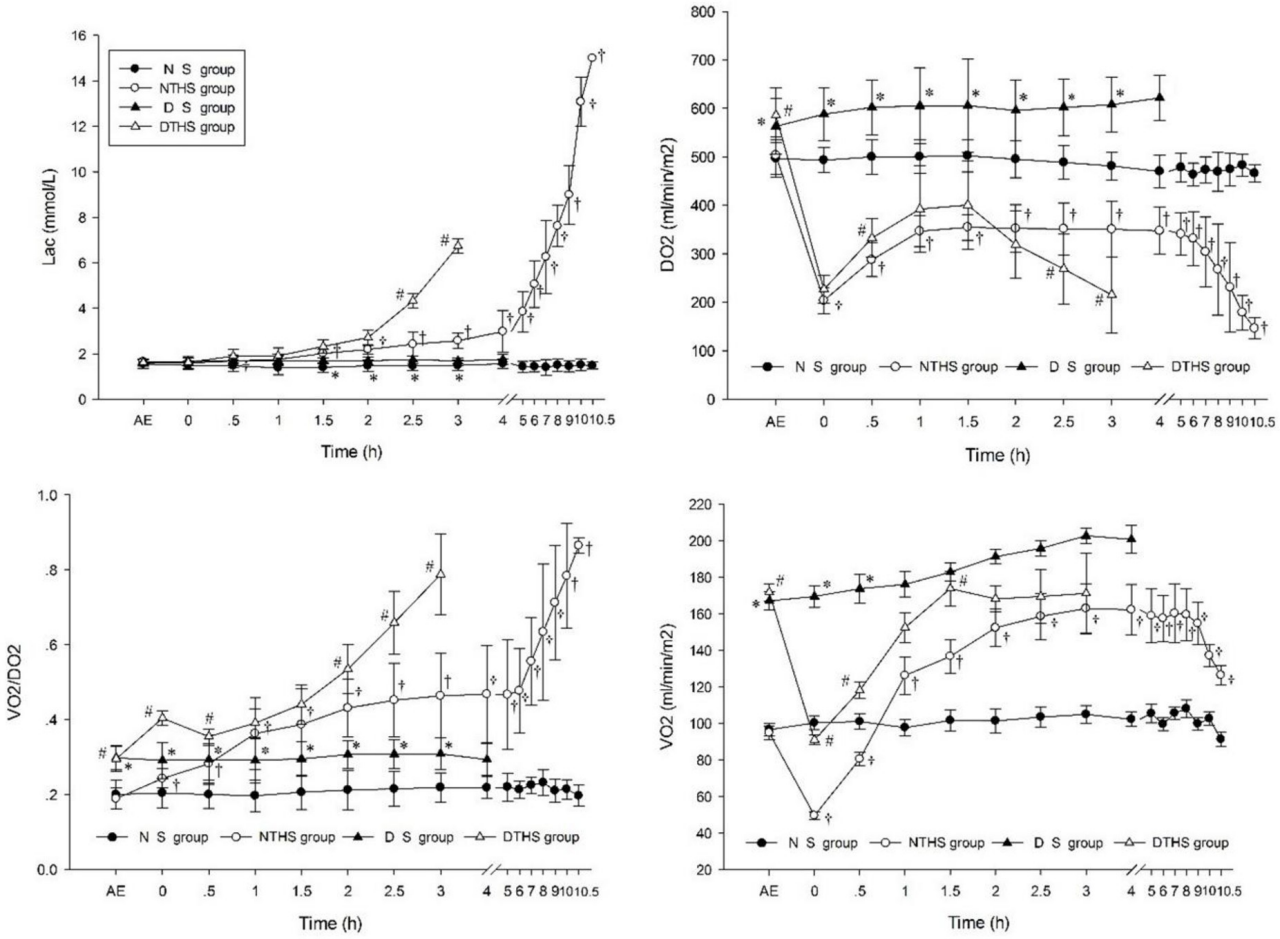
Metabolic effect of oxygen in different groups. BE, before exposure; AE, after exposure; DO_2_, oxygen delivery; VO_2_, oxygen consumption. Values are shown as mean ± SEM. P<0.05 was considered statistically significant. ^†^P, ^#^P, and *P denote statistically significant differences for NS vs. NTHS, DTHS vs. NTHS, and DTHS vs. DS, respectively.

After exposure to the dry-heat environment, VO_2_, DO_2_, and oxygen extraction rate [(O_2_ER) = VO_2_/DO_2_] were significantly higher in the DS and DTHS groups than in the normal temperature environment groups, while DO_2_ and VO_2_ initiated the rising, stabilizing, and declining of MAP, CO, and CI until death in the NTHS and DTHS groups. A steady increase was shown in VO_2_ for the DS group. Lac and O_2_ER increased for the NTHS and DTHS groups, with the increase occurring earlier and quicker for the DTHS group (Fig 6).

After environmental exposure, the organ injury indicators for each group showed no significant differences. No significant differences were observed after exposure (AE) or until the end of the experiment in the NS group. There were no significant differences after a long period (about 2 h) following the test in the NTHS and DTHS groups, except for AST levels. The NTHS group demonstrated a gradual increase in all indicators until animal death, and all indicators were several times higher AE to death. The DTHS group indicators also gradually increased, but the change was small (S1 Table).

TNF-α, IL-6, and IL-10 levels were significantly higher in the DS and DTHS groups after exposure to the dry-heat environment than in the groups exposed to the normal temperature environment. After experiment started, there was an overall increase in the concentrations of inflammatory factors for each group. In a few groups, there was a decrease in inflammatory factors at time 0 h. TNF-α reached peak value first of all the cytokines and it peaked at 6 h for the NTHS group and at 1 h for the DTHS group. IL-6 achieved the greatest increase but it did not peak until animal death. The changes in factors were always observed the earliest and fastest in the DTHS group (Table 1).

**Table 1:** The concentration of inflammatory cytokines in the NS, NTHS, DS, and DTHS groups.

Group	AE	0 h	1 h	2 h	3 h	6 h	10 h
TNF-α (pg/ml)							
NS	23.7±6.8	24.7±10.5	25.3±7.8	26.3±7.1	27.3±12.4	28.3±8.6	30.5±15.4
NTHS	25.1±9.9	23.6±12.9	32.8±10.9	34.3±15.0	34.1±15.1	68.0±19.4^a^	35.2±18.7
DS	37.2±17.9^d^	39.9±12.8	40.4±15.5^b^	41.9±16.1	42.2±10.8	-	-
DTHS	40.4±10.0^d^	38.6±7.4^c^	66.9±14.6^c^	53.5±11.4^c^	35.6±8.1	-	-
IL-1β (pg/ml)							
NS	62.9±15.3	64.7±20.7	61.6±21.2	54.6±21.4	65.6±20.8	63.5±15.6	68.4±6.9
NTHS	68.9±16.8	63.2±19.7	78.1±13.5^a^	85.6±23.4^a^	97.6±18.0^a^	157.8±30.9^a^	122.9±40.4^a^
DS	79.5±13.3	83.8±19.4	85.8±13.2^b^	88.7±19.4^b^	96.3±17.5	-	-
DTHS	84.7±22.8	99.8±14.7^c^	149.1±21.6^c^	157.8±19.7^c^	95.1±19.3	-	-
IL-6 (pg/ml)							
NS	34.5±14.5	38.7±10.5	41.3±15.9	42.4±13.9	50.6±14.7	114.8±19.1	152.9±24.3
NTHS	38.1±18.2	42.8±16.9	54.0±17.3	59.6±24.8	95.9±21.8^a^	505.4±34.7^a^	826.4±28.0^a^
DS	90.6±20.5^d^	110.1±20.0	114.4±27.5	124.8±19.4^b^	139.4±23.8^b^	-	-
DTHS	91.7±18.5^d^	101.4±19.4^c^	121.4±19.2^c^	186.4±27.9^c^	354.8±27.1^c^	-	-
IL-10 (pg/ml)							
NS	35.7±13.3	33.8±15.6	35.6±16.3	40.8±16.0	45.7±22.9	70.0±21.3	75.8±15.1
NTHS	34.8±12.0	26.4±11.2	47.7±25.5	69.9±23.2^a^	114.2±25.2^a^	205.8±22.7^a^	242.8±21.1^a^
DS	56.5±25.3^d^	60.0±27.8	68.7±27.8^b^	83.6±10.8^b^	86.9±14.6	-	-
DTHS	54.5±23.9^d^	43.3±24.9	190.5±28.3^c^	126.8±19.7^c^	77.2±10.6^c^	-	-

AE, after exposure. Values are shown as mean ± SEM. P<0.05 was considered statistically significant. ^a^P, ^b^P, and ^c^P denote a statistically significant difference for NS vs. NTHS, DTHS vs. DS, and DTHS vs. NTHS at the same time point, respectively;

^d^P denotes dry-heat temperature environment vs. normal environment after exposure.

## Discussion

An abdominal closed liver injury does not accurately control the severity of liver damage and the amount of bleeding. To accurately control the degree of liver damage and to improve the comparability of the experiment, all groups underwent laparotomy. Then, the THS groups underwent precise localization and quantitative resection of the liver. While these can all be classified as trauma, the trauma caused by the laparotomy was not the object of our research. However, this trauma has some effects on the body's function (e.g., inflammatory factors). Furthermore, this is an anesthesia model, and anesthesia has some effect on animal metabolism and hemodynamics; however, from the perspective of ethics and experimental compliance, we must administer anesthesia.

In environmental and/or physiological conditions where heat gain outweighs heat loss, internal body temperature increases [20]. Ambient temperature represents the most determinant factor for heat exchange and thermal stress strongly influences thermoregulatory mechanisms [21]. Therefore, the temperature of the DS and DTHS groups continuously increased. When the body temperature is increased by approximately 3°C above normal, it will show severely strained physiology and may even result in death [22]. In fact, an increase of 0.3°C will initiate a heat dissipation mechanism [23]. In addition to heat stress, the DTHS group experienced massive blood loss, which may lead to reduced hypothalamic blood supply and subsequent temperature regulation disorder. This may explain why the temperature in the DTHS group increased faster and earlier.

High fever and hypovolemia often develop simultaneously in a thermal environment [24]. Passive elevation of the body temperature induces blood flow redistribution to the skin [25] that leads to blood decline in central and other organs. Therefore, the MAP in the DS and DTHS groups declined slightly after exposure to a dry-heat environment. This stress activates the neuro-endocrine system, which significantly increases HR and will elevate CO and CI in this phase. Hypovolemic and traumatic bleeding lead to decreased intravascular blood volume, inducing hypovolemic shock. Regardless of the environment, following a hemorrhagic shock, physiological hemodynamic responses such as MAP, CO, and CI are similar in that they compensate, stabilize (plateau stage, PS), and decompensate. However, these stages will be shortened in a dry-heat environment and the PS is extremely short. Positive inotropic action is triggered by heat stress acting alone or in combination with hemorrhagic shock, and this leads to increased HR and myocardial contractility [20]. This was the main reason for the early and rapid increase in HR in the DTHS group. However, this compensatory positive inotropic action is limited, and earlier and stronger consumption may be the main reason for the extremely short PS in the DTHS group.

Dynamic equilibrium of supply and demand of normal physiological activity is dependent upon tissue oxygen metabolism. Usually, levels of DO_2_ should be higher than or equal to levels of VO_2_ [26]. Certain conditions and various disease states can however lead to reduced DO_2_ and (or) increased VO_2_, which interferes with the balance of DO_2_ and VO_2_, leading to oxygen debt occurrence. This in turn leads to tissue and organ damage and may even result in organ failure or death [27]. In the present study, DO_2_, VO_2_, and O_2_ER were higher in those exposed to the dry-heat environment than in those exposed to the normal temperature environment, suggesting that dry-heat environment exposure could increase oxygen metabolism. Therefore, the dry-heat environment probably caused the piglets in the DTHS group to experience rapid exhaustion of their limited oxygen content, and this may be one of the essential reasons for the weak compensation and rapid process of the disease.

Arterial blood lactate is considered an important reference for indicating oxygen deficiency [28], but there is a time difference in the occurrence of tissue lactic acid and circulating lactic acid. In this study, DO_2_, VO_2_, and O_2_ER, but not lactic acid, were significantly higher in the dry-heat groups than in the normal temperature groups at the initial stage. Therefore, DO_2,_ VO_2,_ and O_2_ER can be used as indicators in the early assessment and monitoring of oxygen metabolism for THS in dry-heat environments. Arterial lactic acid concentration is often considered a sensitive indicator of the severity of shock [26, 29]. In the present study, lactic acid had a marked increase at 0.5 h, followed by a progressive and rapid increase in the DTHS group. This suggests that arterial lactic acid can be used as a sensitive indicator for the monitoring and evaluation of oxygen metabolism and the severity of THS in dry-heat environments.

Trauma and/or hemorrhage-induced hypotension significantly increase proinflammatory cytokine production [30, 31]. The inflammatory factors such as IL-6 and IL-10 showed a gradual increase in the NS and NTHS groups. However, the inflammatory factors were significantly higher in the DS group than in the NS group, indicating that the heat stress initiated an immune response. Metabolic acidosis is rapidly activated in response to a variety of inflammatory signals, such as TNF-α [32–34]; TNF-α plays a decisive role in the initiation of inflammation [35, 36]. In this study, rapidly increasing lactic acid and heat stress in the DTHS group were important factors associated with the early-activated release of TNF-α. In other words, heat stress and THS activated the body's immune response mechanism earlier. This suggests that the inflammatory injury resulting from THS occurs early in a dry-heat environment, and that the cascade reaction will be faster than in a normal temperature environment.

Metabolic acidosis leads to Na+/H+ exchanger (NHE1) activation and subsequent [Ca2+] independent cellular/organ injury [37]. Furthermore, extracellular acidification induces neutrophil activation, leading to end-organ dysfunction [38]; in addition, hemorrhagic shock-induces tissue hypoxia, which then causes cell or organ oxidative and nitrosative stress [28], and unrestrained immune activity can cause cell or organ injury [39, 40]. Immune cells release or express a variety of soluble mediators (such as IL-10) that can lead to cell death via apoptosis [40]. As previously mentioned, THS in a dry-heat environment resulted in higher metabolic activity, and severe hypoxia and acidosis occurred early. These factors can cause severe damage to cells and organs as our previous experiments confirmed this [7]. However, in the present study, the indicators of organ damage in the blood were not significantly expressed (S1 Table). This may be due to insufficient blood circulation caused by hemorrhagic shock, reduced tissue perfusion, coupled with dry heat environment, peripheral blood vessel expansion caused by heat dissipation of the body, exacerbating insufficient tissue perfusion so probably lead to they were not released into the blood, but we suspect that organ damage may be earlier and more extensive. This is one area our team will be researching in the future.

In conclusion, this is the first study to successfully establish a THS model in swine in a desert dry-heat environment and compare the dynamic pathophysiological processes. Our findings will inform the development of treatment recommendations and countermeasures to reduce mortality and improve prognosis.

## Supporting information

S1 Table Organ function in the NS, NTHS, DS, and DTHS groups. (DOCX)
