# Supplementary material for: Swine hemorrhagic shock model and pathophysiological changes in a desert dry-heat environment
Source: PLoS One. 2021 Jan 5;16(1):e0244727. doi: 10.1371/journal.pone.0244727 (PMC7785222; doi:10.1371/journal.pone.0244727)
Supplement: S1 Table — (DOCX) [file pone.0244727.s001.docx]

**S1 Table.** **Organ function in the NS, NTHS, DS, and DTHS groups**

| Group | AE | 0 h | 1 h | 2 h | 3 h | 8 h | 10 h |  |
| --- | --- | --- | --- | --- | --- | --- | --- | --- |
|  | ALT (U/L) | | | | | | | |
| NS | 47.30±7.05 | 49.20±6.13 | 49.70±7.23 | 50.70±6.89 | 51.20±6.68 | 51.40±7.25 | 51.40±7.09 |  |
| NTHS | 51.80±10.30 | 47.00±9.61 | 43.90±9.13 | 44.40±8.52 | 44.80±7.39 | 61.00±9.93^a^ | 73.10±11.49^a^ |  |
| DS | 52.30±6.48 | 49.40±4.84 | 49.80±6.37 | 49.80±6.65 | 53.50±4.76 |  |  |  |
| DTHS | 49.90±10.31 | 44.80±5.49 | 45.00±9.25 | 45.90±10.31 | 47.00±11.00 |  |  |  |
|  | AST (U/L) | | | | | | | |
| NS | 34.40±6.82 | 35.90±7.72 | 36.80±7.34 | 37.50±7.90 | 38.10±8.35 | 39.20±9.74 | 41.80±9.68 |  |
| NTHS | 32.90±7.66 | 37.50±8.50 | 39.90±9.08 | 45.20±10.78 | 49.30±12.3^a^ | 186.20±153.89^a^ | 435.20±168.27^a^ |  |
| DS | 31.90±4.41 | 31.60±7.27^b^ | 32.90±7.71^b^ | 34.30±8.44^b^ | 34.80±10.00^b^ |  |  |  |
| DTHS | 34.30±10.63 | 45.10±6.31^c^ | 59.60±12.04^c^ | 75.50±13.67^c^ | 109.40±12.88^c^ |  |  |  |
|  | BUN (mg/dl) | | | | | | | |
| NS | 3.80±0.73 | 4.70±0.89 | 5.40±1.03 | 6.10±1.12 | 7.10±1.30 | 11.30±1.95 | 13.00±1.98 |  |
| NTHS | 4.10±0.43 | 4.80±0.85 | 5.50±0.89 | 6.60±1.11 | 7.80±1.24 | 12.80±1.87 | 14.40±0.78 |  |
| DS | 3.80±0.31 | 3.90±0.33 | 4.40±0.54 | 4.90±0.55^b^ | 5.30±0.49^b^ |  |  |  |
| DTHS | 3.80±0.28 | 4.30±0.38 | 4.70±0.43^c^ | 5.90±0.49 | 7.10±0.38 |  |  |  |
|  | CREA (μmol/L) | | | | | | | |
| NS | 94.40±7.84 | 95.00±8.32 | 95.20±9.00 | 95.80±6.15 | 96.90±6.28 | 98.10±5.89 | 99.50±7.09 |  |
| NTHS | 97.10±6.46 | 101.40±7.54^a^ | 112.20±8.38^a^ | 124.20±10.93^a^ | 141.10±12.73^a^ | 202.50±18.04^a^ | 234.40±27.10^a^ |  |
| DS | 96.20±5.71 | 101.20±6.23 | 109.80±5.04 | 119.20±5.64^b^ | 130.20±6.31^b^ |  |  |  |
| DTHS | 98.70±1.75 | 99.90±4.02 | 114.60±7.20 | 142.10±11.71^c^ | 183.00±9.10^c^ |  |  |  |
|  | CK  (mmol/L) | | | | | | | |
| NS | 860.40±239.31 | 937.80±381.28 | 893.40±226.04 | 981.30±204.09 | 972.20±195.69 | 902.00±229.48 | 888.80±236.96 |  |
| NTHS | 993.70±249.93 | 823.20±168.90 | 1118.90±308.20 | 1313.00±308.53 | 1556.40±670.32 | 3557.20±886.30 | 4362.40±840.27 |  |
| DS | 890.50±297.92 | 987.50±371.92 | 1064.10±380.03 | 1185.90±513.63 | 1259.30±441.14 |  |  |  |
| DTHS | 844.10±272.05 | 768.10±233.91 | 1070.60±410.90 | 1341.30±385.44 | 1717.5±533.91^c^ |  |  |  |

Values are presented as mean±standard deviation. CREA, creatinine; BUN, blood urea nitrogen; CK, creatine kinase. Values are show as mean±SEM. a, b, and c denote a statistically significant difference for NS vs. NTHS, DTHS vs. DS, and DTHS vs. respectively, NTHS at the same time point.
